# Preliminary landscape analysis of deep tomographic imaging patents

**DOI:** 10.1186/s42492-023-00130-x

**Published:** 2023-01-23

**Authors:** Qingsong Yang, Donna L. Lizotte, Wenxiang Cong, Ge Wang

**Affiliations:** 1 Rensselaer Polytechnic Institute, Troy, NY 12180 USA; 2 Murtha Cullina LLP, Boston, MA 02110 USA

**Keywords:** Artificial intelligence, Machine learning, Deep learning, Medical imaging, Tomography, Image reconstruction

## Abstract

Over recent years, the importance of the patent literature has become increasingly more recognized in the academic setting. In the context of artificial intelligence, deep learning, and data sciences, patents are relevant to not only industry but also academe and other communities. In this article, we focus on deep tomographic imaging and perform a preliminary landscape analysis of the related patent literature. Our search tool is *PatSeer*. Our patent bibliometric data is summarized in various figures and tables. In particular, we qualitatively analyze key deep tomographic patent literature.

## Introduction

With the advances of artificial intelligence (AI), deep learning (DL) has emerged as a mainstream approach with successful applications in many areas. Since 2016, deep reconstruction or deep imaging methods have been actively developed, especially in the field of medical imaging [1–3]. Promising results on medical imaging are widely reported on diverse topics ranging from data acquisition and processing [4], image reconstruction and enhancement [5, 6] to radiomics and health analytics [7], and more. Clearly, AI/DL is paving an exciting way to improve or innovate medical imaging devices, and diagnostic and therapeutic procedures. With rigorous and systematic assessment and regulation, AI imaging software and devices may assist or compete effectively with radiologists, eventually transforming the current model of medical and healthcare practice in various aspects.

**Fig. 1:**
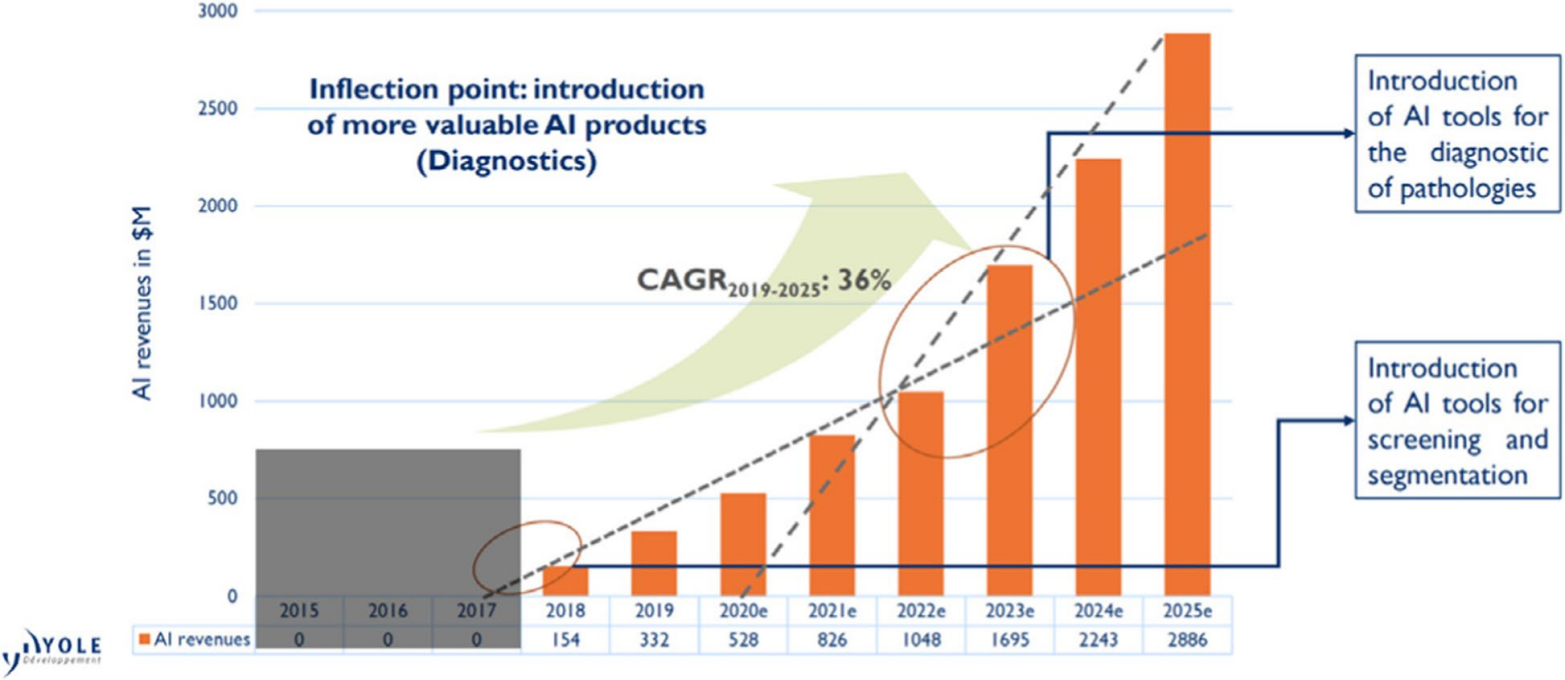
AI revenue of the medical imaging companies from 2015 to 2025 (adapted from www.yolegroup.com)

According to a 2020 press release from the Yole Group (https://www.yolegroup.com/press-release/medical-imaging-artificial-intelligence-changes-the-rules/), “*AI has the potential to change all of our diagnostics and treatment procedures to enable more personalized and effective medicine.” “At Yole, we estimate the total market in 2025 for software generated revenues through the sale of AI tools will reach US$2.9 billion with a 36% CAGR (Compound Annual Growth Rate) between 2019 and 2025 (Fig. **1**). These revenues can be shared between the main applications including improved image capture, noise reduction, image reconstruction, screening, diagnostic and treatment planning*.”

Given the huge commercial potential of AI-based imaging technologies, intellectual property plays an increasingly important role in the imaging industry, research and user communities. In this context, for researchers and developers, patent landscape analysis and literature review are indispensable. To understand the landscape of AI-based deep tomographic imaging technologies that promise to be clinically relevant, we are motivated to survey relevant patent literature over the past decade or so. As used herein, “patent literature” includes issued patents and published patent applications (i.e., pre-grant patent application publications). As further used herein, “patent documents” (“PGPubs”) are also used to mean patent literature.

The rest of this paper is organized as follows. In the second section, we present our methodology. Our main patent search tool is *PatSeer* [8]. Our search strategy and analysis methods are described in this section. In the third section, we present our *PatSeer* search results in figures and tables. While the third section is largely data-driven, in the fourth section we analyze key deep tomographic patent documents aided by citation analysis. In the last section, we discuss relevant issues and conclude the paper.

## Methods

Over recent years, the importance of patent literature has become more recognized in the academic setting, as evidenced by the establishment of the National Academy of Inventors in 2010. In the context of AI, DL, and data science, patents are relevant to not only industry but also academe and other communities. To perform our landscape analysis of the patent literature on deep tomographic imaging, we used the patent search and analysis tool *PatSeer*. We note that other patent search and analysis tools are also available including, but not limited to, AcclaimIP (available from Anaqua), LexisNexis TotalPatent One®, etc.

Specifically, the tool we used is *PatSeer ProX*, developed by Gridlogics. *PatSeer ProX* includes big-data analytic methods and performs relatively fast through, in our opinion, a very user-friendly interface. We find that the search rules used by *PatSeer ProX* are like those used by Scopus. According to the *PatSeer* website, the *PatSeer ProX* patent database covers more than 136-million patent publications, and 96-million full-text records. Furthermore, *PatSeer* maintains a scalable big-data platform with AI-based semi-automated algorithms to process and analyze raw data from over 300 sources.

**Fig. 2:**
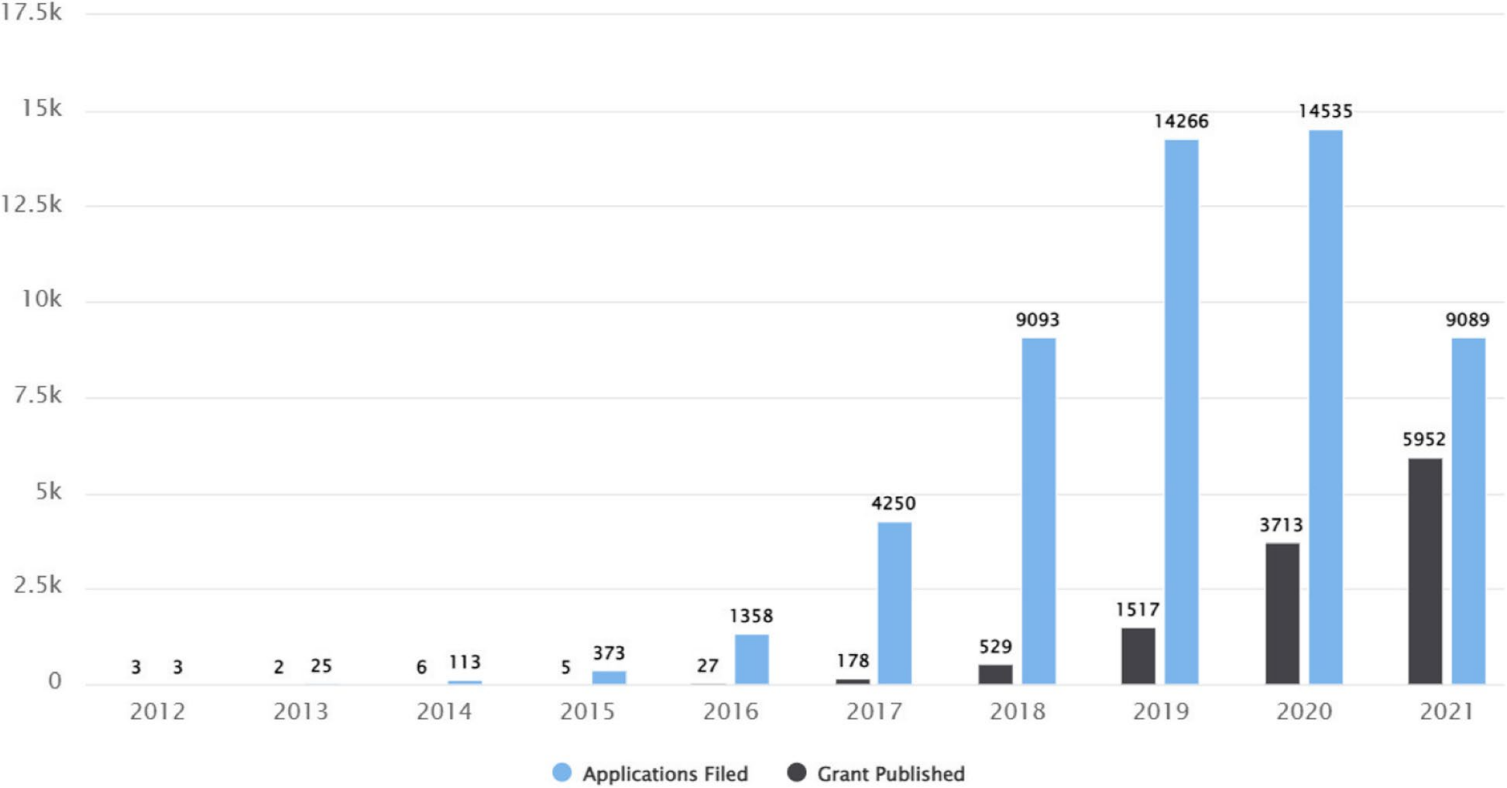
Patenting trend in the field of DL. The chart shows filing and grant data, with the applications being plotted by filing date and the grants by publication date

**Fig. 3:**
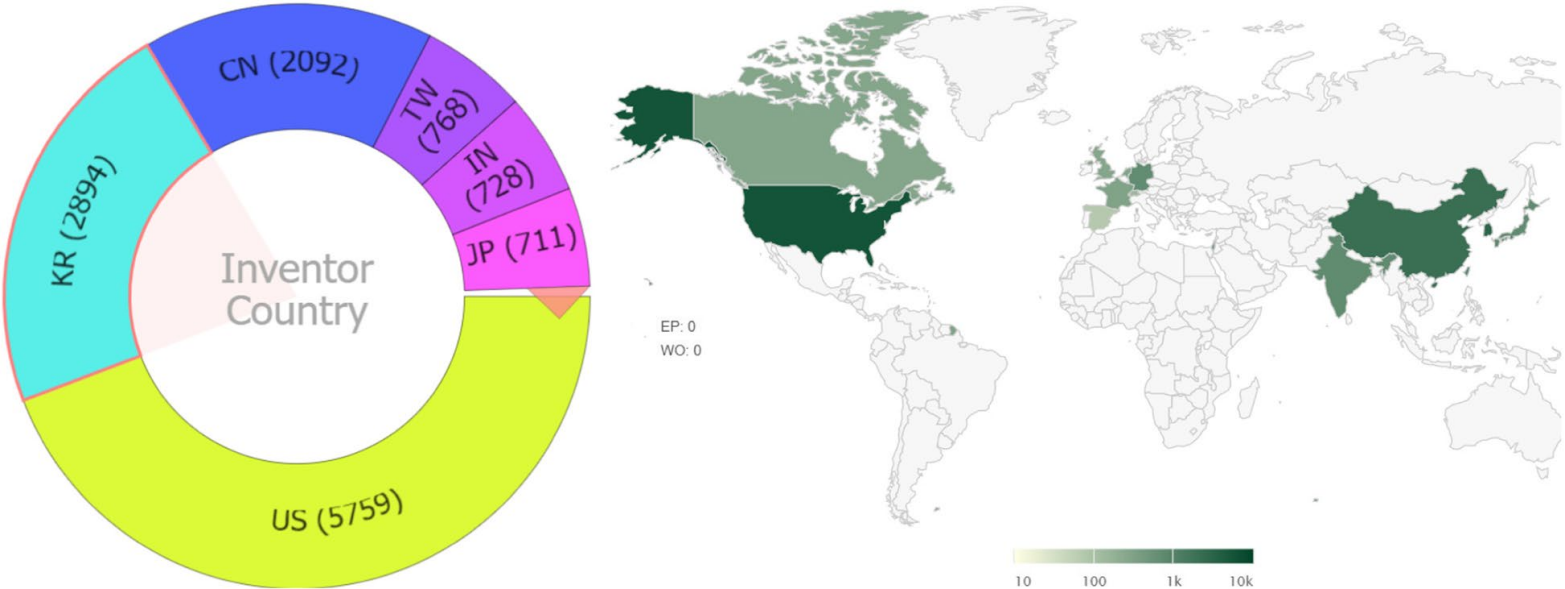
Distribution of inventors by country of residence. The left chart shows the number of records by the country of residence of inventors. The right chart shows the number of records by the country where the application was filed. Note that a record is included in each unique country (the same record may be repeatedly counted)

As an illustration, with the “deep learning” as the search phrase in the title, abstract and claim fields from January 1, 2010 to the end of 2021 (*TAC:("deep learning") AND PBD:[2010–01-01 TO 2021–12-31]*), Figs. 2 and 3 were generated in seconds.

## Results

### Visualization of Patents on Deep Tomographic Imaging

In this section, we focus on our patent search results on AI-based medical imaging techniques. Initially, we performed keyword-based searches in the title, abstract and claim fields but we obtained quite many irrelevant results. For example, the inclusion of claims included French PGPub FR2733596B1 [9] which only mentions ‘Ultrasound’ in the claims. As another example, International PGPub WO0124700A1 [10] is for fingerprint detection. As a result, our searches were limited to the title and abstract fields only. After further deliberation, we defined the following search expression: TA:(("machine learning" or "deep learning" or "deep nets" or "neural network" or "deep network" or "deep neural network" or "artificial intelligence") and ((raw data process* or "k-space data" or "tomographic data" or "sinogram" or image reconstruct* or post-process*) or ("image quality" or "artifact reduction" or “low dose scan” or “fast scan” or “under sample” or “noise reduction”)) and (medical imag* or tomograph* or "CT" or "computed tomography" or "PET" or "positron emission tomography" or "SPECT" or "single photon emission computed tomography" or "nuclear imaging" or "MRI" or "magnetic resonance" or "Ultrasonography" or "ultrasound" or "optical coherence tomography" or "OCT") and not ("display apparatus" or assess*)), where TA means in the title and abstract fields. Note that the filter function, not ("display apparatus" or assess*), was empirically added to exclude irrelevant hits; for example, DE69031523D1 [11] matches the other parts of the overall expression but only describes a method to optimize a display window, and US8086007B2 [12] describes an image quality assessment method. In total, this search yielded 757 records.

**Fig. 4:**
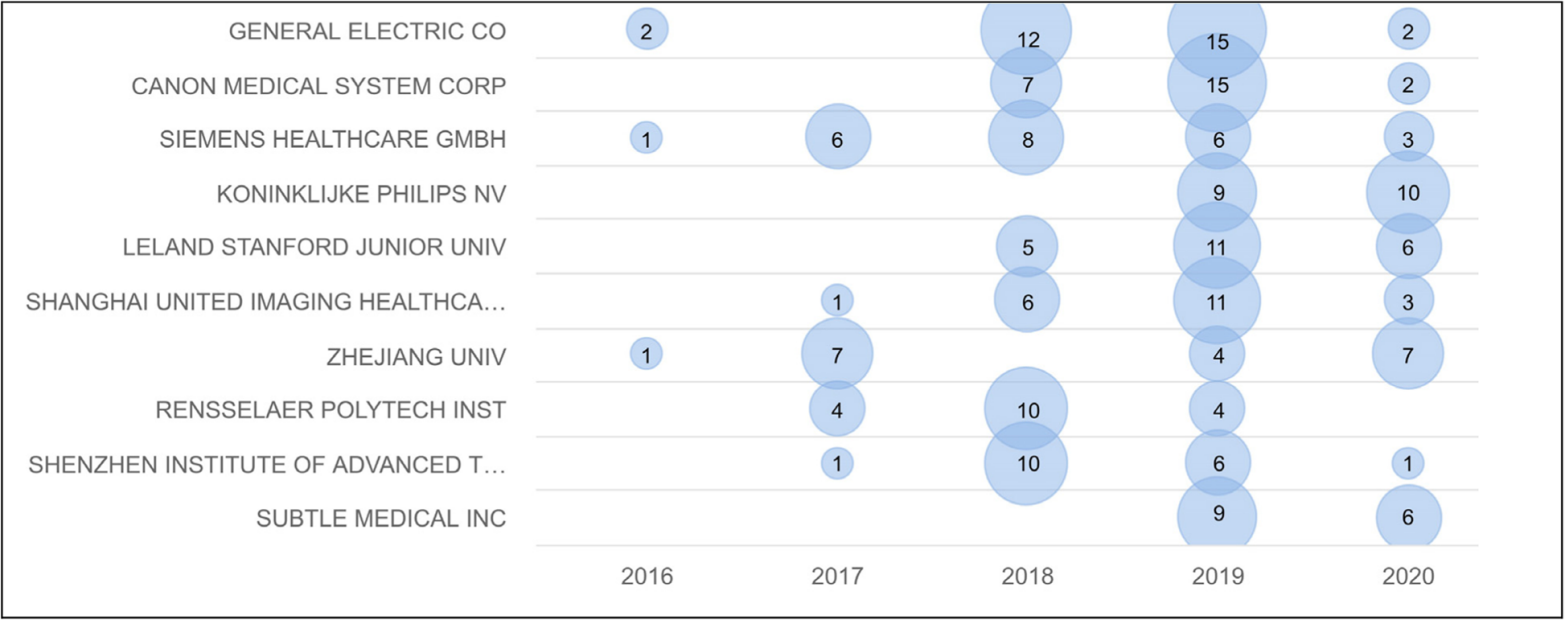
Innovation timeline by original assignee vs application date, where innovation intensity is shown by the number of assigned patent applications per assignee per year

**Fig. 5:**
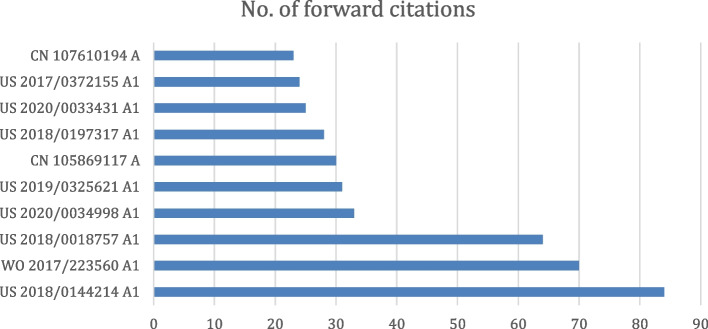
Top 10 most cited issued patents and PGPubs in the domain of interest

Figure 4 summarizes the numbers of patents per application year grouped by the original assignee. Figure 5 lists the top 10 most cited patents in this domain. A few examples are described here. PGPub US20180018757A1 describes a method to improve the quality of the projection data using machine learning (ML) models. International PGPub WO2017223560A1 [13] describes ML-based tomographic/tomosynthetic techniques that use a neural network that includes more than three layers and can be applied to either raw data or initial image domains. PGPub US2020034998A1 [14] is an example of DL for MRI imaging, where a neural network model is used with data consistency.

To assess the quality of an issued patent or published patent application quantitatively, *PatSeer* provides a 360° Quality metric, which is a weighted average of 4 contributing scores. The contributing scores include Citation Quality (CQ), Market Quality (MQ), Legal Quality (LQ) and Document Quality (DQ) [8]. CQ considers the number, recency and type of forward citations of a patent document. MQ is based on the patent family’s global market coverage. LQ reflects how aggressive the company protects the patent family. DQ measures the intrinsic quality of a patent based on its structural elements. Then, the portfolio value index is defined as the sum of the 360 Quality scores of all the patent families present in a portfolio, which, according to Gridlogics, is roughly proportional to the overall realizable value of the portfolio.

**Fig. 6:**
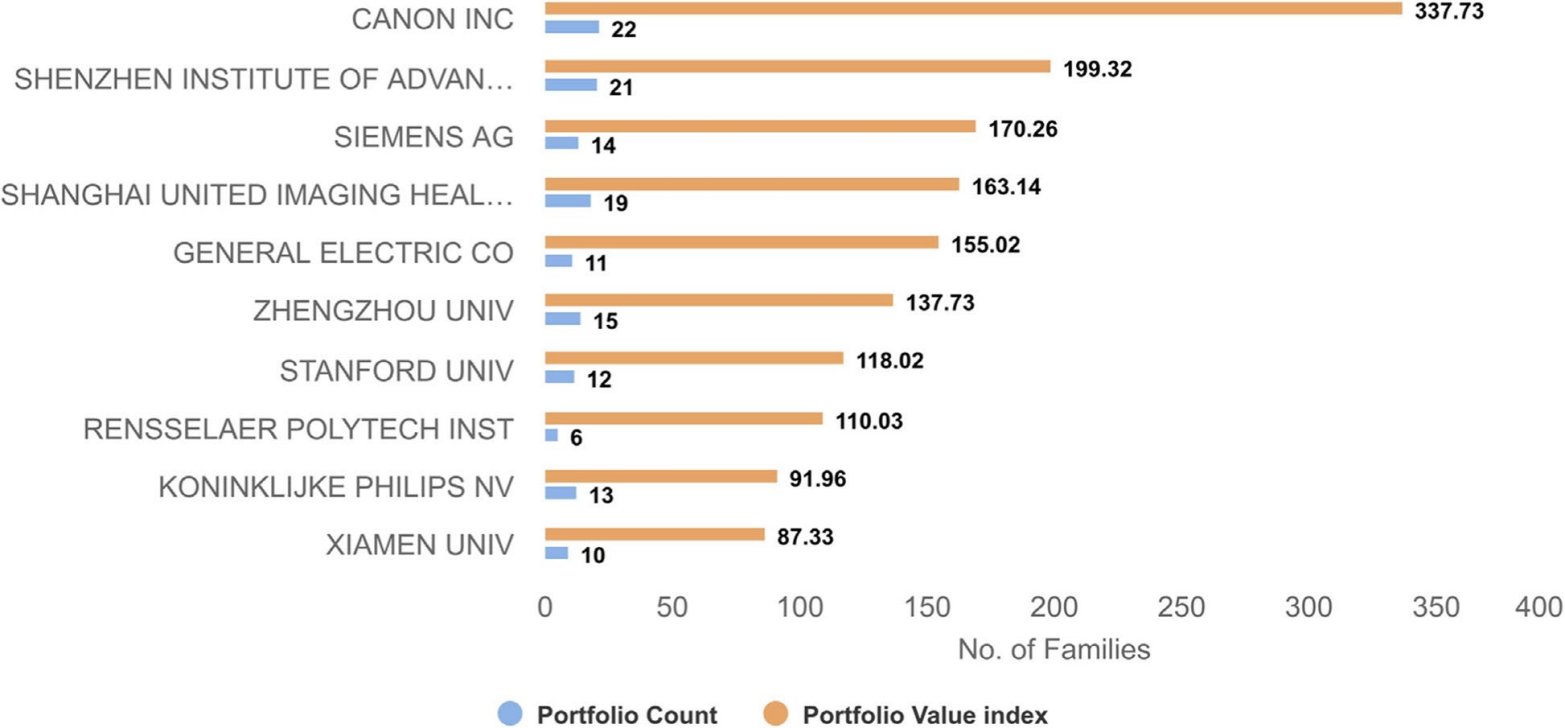
Most valuable portfolios (benchmarked by PatSeer’s 360^0^ score) showing top players with the highest portfolio value index

Figure 6 shows the most valuable portfolios, according to the *PatSeer* Quality metric. Among them, Generic Electric Co (GE), Canon Inc and Siemens AG hold the most valuable portfolios as industry leaders, while Rensselaer Polytechnic Institute (RPI), Zhengzhou University and Stanford University are the academic leaders.

## Analysis of Patents on Deep Tomographic Imaging

We work in the medical imaging field with diverse interests but with an emphasis on computed tomography (CT). Given the broad view presented in the preceding section, in this section we analyze further deep CT reconstruction patent literature and other related technologies. This analysis provides a unique perspective that will help us plan future research and development activities. In the following sub-sections, we analyze patent literature based on specific applications and select representative patent documents for analysis. Tables 1, 2 and 3 list related patent documents and further include brief comments.

**Table 1:** Representative patent literature for image reconstruction using AI technology

No	Title	Comments	Owner	Priority date
WO2017223560A1	Tomographic image reconstruction via ML	A ML method is proposed to improve the quality of tomographic images by applying ML models on raw data, processed data, or an intermediate image to reduce noise and artifacts.	RPI	06–24-16
WO2018126396A1	DL based estimation of data for use in tomographic reconstruction	Trained neural network is used to estimate various types of missing projection data.	GE	01–05-17
US2018197317A1	DL based acceleration for iterative tomographic reconstruction	A DL technique is used to accelerate iterative reconstruction of images.	GE	01–06-17
US2019102916A1	Systems and methods for DL-based image reconstruction	A method includes acquiring a set of imaging projections data, identifying a voxel to be reconstructed, receiving a trained regression model, and reconstructing the voxel.	GE	09–29-17
US2021074033A1	DL-based data rescue in emission tomography medical imaging	An emission image is generated from poor quality emission data. A machine-learned model is used to recover information related to the data.	Siemens	09–09-19
US2017372193A1	Image correction using a deep generative machine-learning model	A deep-learnt generative model is used as a regularizer in an inverse solution with a physics model of the degradation behavior of the imaging system. The generative model is trained from good images, so difficulty gathering problem-specific training data may be avoided or reduced.	Siemens	06–23-16
US2020311490A1	Apparatus and method for sinogram restoration in CT using adaptive filtering with DL	A method is proposed to train a DL network to optimize the convolution kernel of an adaptive filter that is applied in the data domain. The input data can be sinograms generated by a low-dose CT scan, and the target data generated by a high-dose CT scan.	Canon	04–01-19
US2021012541A1	Apparatus and method using DL to improve analytical tomographic image reconstruction	A method is proposed to improve the image quality of images generated by analytical reconstruction of a CT image. This improved image quality results from a DL network that is used to filter a sinogram before back projection but after the sinogram has been filtered using a ramp filter or other reconstruction kernel.	Canon	07–11-19
US2021192809A1	Tomographic image reconstruction using AI engines	A method includes obtaining two-dimensional (2D) projection data and processing the 2D projection data using the AI engine. AI engine may involve: generating 2D feature data by processing the 2D projection data using the multiple first processing layers, reconstructing first three-dimensional (3D) feature volume data from the 2D feature data using the back-projection module; and generating second 3D feature volume data by processing the first 3D feature volume data using the multiple second processing layers.	Varian Medical System	12–20-19
EP3367329A1	Denoising medical images by learning sparse image representations with a deep unfolding approach	Method is to learn sparse image representations with deep unfolding and applying the machine learnt network medical image denoising.	Siemens	02–22-17
WO2019060843A1	Image reconstruction using ML regularizers	A method for reconstructing an image of a target object using an iterative reconstruction technique can use a ML model as a regularization filter.	Nview Medical Inc	09–22-17
US2021118204A1	Method for reconstructing incomplete data of X-ray absorption contrast computed tomography based on DL	A DL-based method is proposed using a filtered back projection (FBP) algorithm to obtain an initial reconstructed image; forward projecting the initial reconstructed image to obtain artifact-contaminated complete projection sequences; using a DL technique to process the artifact-contaminated projection sequences to obtain artifact-free projection sequences; using the FBP algorithm to reconstruct the artifact-free projection sequences to obtain a final reconstructed image.	Beihang of University	10–18-19
US2021118200A1	Systems and methods for training ML algorithms for inverse problems without fully sampled reference data	A self-supervised training of ML algorithm is proposed for reconstruction in inverse problems. A physics-based ML reconstruction can be trained without requiring fully-sampled training data.	University of Minnesota	10–21-19
US2018018757A1	Transforming projection data in tomography by means of ML	A method is proposed to use a ML model to outputs high quality projection data from low quality data.	Suzuki Kenji	07–13-16
WO2018236748A1	DL-assisted image reconstruction for tomographic imaging	An iterative image reconstruction method produces a plurality of intermediate images and to produce the image of the subject. One selected intermediate image from the plurality of intermediate images using a quasi- projection operator. The quasi-projection operator uses a deep-learning model configured to map the at least one selected intermediate image to at least one regularized intermediate image.	University of Washington	06–19-17
EP2890300B1	Supervised ML technique for reduction of radiation dose in computed tomography imaging	A technique is proposed for converting low-dose CT images to higher quality, lower noise images using ML.	University Chicago	08–31-12
WO2019074879A1	Image generation using ML	A ML model is used to convert an input image generated from a computationally efficient algorithm to a final high quality image.	GE	10–11-17
WO2018187020A1	Tomographic reconstruction based on DL	Tomographic images are used as an input to a neural network. More layers of the neural network are used as wavelet filter banks.	GE	04–05-17
US2019104940A1	Apparatus and method for medical image reconstruction using DL for CT image noise and artifacts reduction	A method is proposed to reduce noise and artifacts in reconstructed medical images using a DL network.	Canon	10–06-17

**Table 2:** Representative patent literature for motion compensation, metal artifacts, and material decomposition

No	Title	Comments	Owner	Priority date
US2020273215A1	Monochromatic CT image reconstruction from current-integrating data via ML	A neural network is configured to learn a nonlinear mapping function to map from a CT image reconstructed from a single spectral current-integrating projection data collected in a current-integrating X-ray detector to an image reconstructed from a virtual monochromatic projection data at a pre-specified kVp energy level. The technique realizes monochromatic CT imaging and overcomes the beam hardening problem.	RPI	09–26-17
US2020196973A1	Apparatus and method for dual-energy CT image reconstruction using sparse kVp-switching and DL	A neural network is trained to suppress artifacts in the reconstructed CT images. Another network is trained to perform image-domain material decomposition from the previous model’s output to correct beam hardening and spatial variations in the X-ray beam.	Canon	12–21-18
US2019130571A1	Method and system for compensating for motion artifacts by means of ML	A ML method is used for motion artifacts compensation.	Siemens	10–27-17
US2019295294A1	Method for processing parameters of a machine-learning method and reconstruction method	A method is proposed for providing a correction dataset for motion correction of a CT image dataset of an object using processing parameters of a machine-learning method.	Siemens	03–23-18
US2019328341A1	System and method for motion estimation using AI in helical computed tomography	A method is proposed for estimating and compensating motion artifacts produced during image reconstruction from helical CT scan data.	Canon	11–16-16
US2021056688A1	Using DL to reduce metal artifacts	An image correction method is proposed by using a neural network to generate a metal artifact image from a CT image; and generating a corrected X-ray image by subtracting the metal artifact image from the original image.	Philips	01–26-18
WO2020033355A1	DL-based method for metal reduction in CT images and applications of same	A deep-learning-based method is proposed for metal artifact reduction in CT images.	Vanderbilt University	08–06-18
WO2019063760A1	DL based scatter correction	A neural network is trained on Monte Carlo simulated imaging data with at least one X-ray photon scattering mechanism to convert the projection data to a scatter free data, which is further used to reconstruct the CT image.	Philips	09–28-17

**Table 3:** Representative patents for radiomics

No	Title	Comments	Owner	Priority date
WO2018220089A1	ML on raw medical imaging data for clinical decision support	A raw diagnostic machine for a medical diagnosis of raw medical imaging data generated by a medical imaging machine without conventional image reconstruction.	Philips	05–31-17
US2021192810A1	Tomographic image analysis using AI engines	A method is proposed by obtaining first 3D feature volume data and processing the first 3D feature volume data using an AI engine that includes multiple first processing layers, an interposing forward-projection module and multiple second processing layers.	Varian Medical System	12–20-19
WO2018232388A1	Systems and methods for integrating tomographic image reconstruction and radiomics using neural networks	A ML framework is used to either integrate tomographic images and radiomics or map tomographic raw data directly to diagnostic information.	RPI	06–16-17
US2020311878A1	Apparatus and method for image reconstruction using feature-aware DL	A method is proposed to use a neural network to perform on a feature-aware reconstruction. A neural network model is trained to perform feature-aware reconstruction using a target image is spatially-dependent degree denoised and artifact suppressed based on the features in the images.	Canon	04–01-19
WO2020214911A1	Method and system for generating attenuation map from single photon emission computed tomography (SPECT) emission data based upon DL	A system is proposed to use ML models to estimate attenuation coefficients from only SPECT emission data.	Yale University	04–19-19
CN111598895A	Method for measuring lung function indexes based on diagnosis images and ML	A method is proposed for measuring lung function indexes based on diagnostic images and ML.	Suzhou Fuyuan Medical Tech	04–14-20

### Tomographic image reconstruction

DL has significant implications for tomographic image reconstruction, as first described in our perspective paper on deep imaging [1]. Our perspective paper presents three specific examples with simulation results; i.e., CT image super-resolution, singogram quality improvement, and CT image denoising. More generally, when a dataset is truncated, for example, in cases of limited-angle, few-view and interior or local reconstruction, or an image is distorted or comprised by artifacts, including metal artifact, scatter artifact, motion artifact and beam-hardening effect, a synergistic combination of conventional tomographic methods and DL-based imaging may enhance image quality and diagnostic performance.

An international patent application (PGPub WO2017223560A1) [13] was filed with a priority date of June 24, 2016, describing tomographic image reconstruction systems and methods based on DL techniques. This PGPub discloses a general framework for image reconstruction from raw data directly or from an intermediate reconstructed image with the DL/ML approach, suggesting a potential for deep tomographic reconstruction to surpass classic reconstruction algorithms.

Another published international patent application (PGPub WO2019074879A1) [15] is directed to image reconstruction with DL/ML. The approach involves a ML model training process and use of the trained model for image generation. The ML model training may use multiple images generated from a single set of tomographic projections or images. The trained model may be used to generate a final image from the projection data, which is a less computationally intensive algorithm. The issued patent US10475214B2 [16] uses ML to solve tomographic reconstruction problems, which are usually in large scale and are space variant while correcting various artifacts. International PGPub WO2018126396A1 [17] presents DL-based raw data correction and estimation for tomographic reconstruction.

International PGPub WO2019060843A1 [18] discloses an image reconstruction method using a ML regularizer. Specifically, an iterative reconstruction technique can incorporate a ML model as a regularization filter for the image reconstruction. International PGPub WO2018236748A1 [19] describes an image reconstruction scheme using DL/ML for tomographic imaging. The method first produces a plurality of intermediate images using iterative reconstruction method and furthermore transforms selected intermediate images using a deep-learning based quasi-projection operator to a regularized intermediate image.

PGPub US2018/0018757A1 [20] discloses a technique for improving projection data via DL/ML. The method transforms projection data from low-quality projection to higher quality, and performs image reconstruction from the improved projection images. A DL model is trained from matched pairs of lower-quality (lower-dose) and corresponding higher-quality (higher-dose) projection data and used to transform lower-quality (lower-dose) projection data to higher-quality (higher-dose) ones after training is finished. PGPub US2020311878A1 [21] describes a feature-aware DL method for image reconstruction. A neural network model is trained to perform feature-aware reconstruction using a target image for spatially-dependent denoising and artifact suppression.

The last technology we comment on is virtual monochromatic CT image reconstruction. International PGPub WO2019067524A1 [22] discloses processing current-integrating data and images via ML to produce virtual monochromatic images. The neural network is configured to learn a nonlinear function to map a CT image reconstructed from projection data collected in a current-integrating X-ray detector to an image reconstructed from a virtual monochromatic projection dataset at a pre-specified kVp energy. The technique realizes monochromatic CT imaging and overcomes the beam hardening problem. PGPub US2020196973A1 [23] discloses an apparatus and method for a dual-energy CT system which uses sparse kVp-switching to collect data and a DL method to reconstruct the image. The deep network is fed with images that include artifacts and is trained to output images with little or no artifacts. Another deep network model can be trained to perform material decomposition from the artifact-free images.

### Artifact reduction

**Fig. 7:**
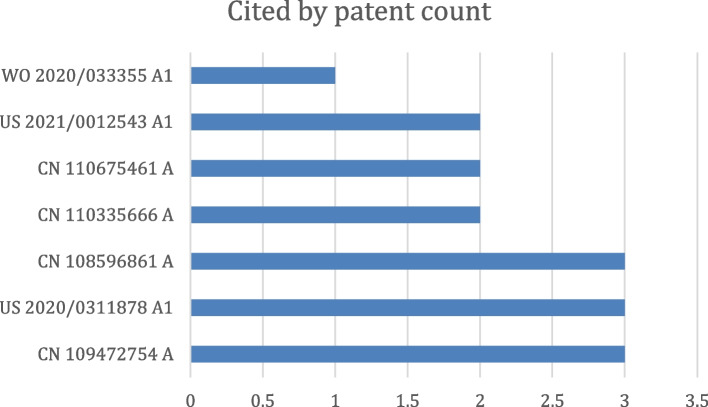
Most often cited patent documents related to CT metal artifact reduction, ranked by the number of forward citations

Metal artifact reduction is one of the remaining problems in the CT field. European PGPub EP3743889A1 [24] discloses using DL to reduce metal artifacts. The neural network model is trained to generate a metal artifact image and generate a new image by subtracting the estimated metal artifacts from the input image. A relatively high number of patent documents target the same problem. Figure 7 lists some of them, ordered according to the number of forward citations.

**Fig. 8:**
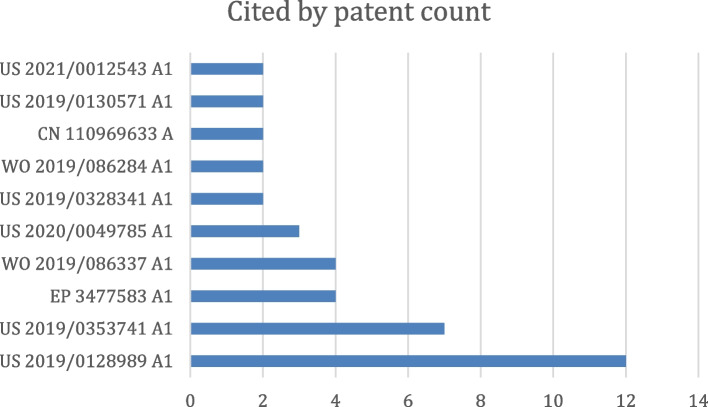
Most cited patent documents on CT motion artifact reduction, ranked by the number of forward citations

The German PGPub DE102017219307B4 and Chinese PGPub CN109727203B [25, 26] describe a system and method for compensating motion artifacts via ML. The technology relates to an automatic compensation method for motion artifacts in a medical image. The technology further relates to a method of automatically identifying motion artifacts, and includes a compensation unit, a learning device, and a controlling device for medical imaging. A number of patent documents address this problem, as shown in Fig. 8, ranked according to the number of forward citations.

**Fig. 9:**
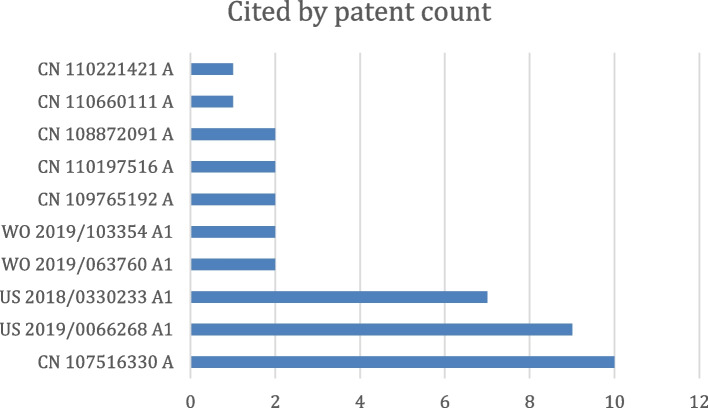
Most cited patent documents on CT/PET scattering correction, ranked by the number of forward citations

The Japanese PGPub (JP2020534929A) [27] presents a DL-based scattering correction for X-ray imaging. A neural network is trained with a Mone Carlo simulated imaging data. This simulation includes at least the X-ray photon scattering mechanism that contaminates CT data with scattering noise. The neural network model learns to remove the scattering noise in the CT data. After CT data are corrected by removing scatters in the projection space, the image reconstruction is performed to generate a scattering-corrected CT image. Scatter correction is also important for positron emission tomography (PET) imaging. A number of patent documents exist related to DL-based scatter correction for either CT or PET, as shown in Fig. 9.

### Image analysis, radiomics and rawdiomics

International PGPub WO2018232388A1 [28] uses neural networks to integrate tomographic image reconstruction and radiomic analysis. CT screening, diagnosis or image analysis tasks are often performed with separate neural networks and algorithms. Integrating these elements into an end-to-end workflow may streamline the whole process and optimize the task-specific performance. As we know, while deep reconstruction is for image formation from raw data, image analysis or radiomics is for image analysis. Thus, the claimed integration of image reconstruction and image analysis is referred to as ‘rawdiomics’, where ‘rawd’ means raw data, and ‘i’ indicates images or informatics. International PGPub WO2018/220089A1 [29] applies DL/ML to raw medical imaging data analysis for clinical decision support. The techniques are intended for medical diagnosis from raw imaging data generated by a medical imaging machine with or without conventional image reconstruction.

## Discussion and conclusions

First of all, we underline that although *PatSeer ProX* has provided useful information, we are still on the learning curve. We are not familiar with all of the functions and terminology of the *PatSeer ProX* tool. As a result, we believe that hidden information in the *PatSeer* dataset can be further mined. While we believe that we have obtained valuable data in this study, omissions and biases are unavoidable, due to the imperfect coverage of the database, dynamic nature of the field, and our limited capabilities. We apologize if we have made any misinterpretations.

Despite any problems possibly existing in this preliminary patent landscape analysis, we have learned significantly, facilitated by *PatSeer ProX.* Clearly, research and development in deep tomographic imaging has a strong momentum, engages both imaging companies and academic groups, and promises lasting impact on the further research and development as well as market and healthcare. Since the field of deep tomographic imaging is relatively young, more results and data are yet to be collected and analyzed to reveal the patent dynamics in terms of licensing, revenue, and translated outcomes. Also, it may be very informative to use *PatSeer ProX,* or similar tools, to identify and track emerging areas of AI-based imaging activities.

In conclusion, we have performed a preliminary landscape analysis on patent literature dedicated to deep tomographic imaging. Using the *PatSeer ProX* tool, we have systematically collected and analyzed relevant bibliometric data, and commented on representative deep tomographic imaging patent documents. Finally, we have discussed several issues and future work on these interesting topics.

## Data Availability

Not applicable

## Abbreviations

AI: Artificial intelligence
ML: Machine learning
DL: Deep learning
CQ: Citation Quality
MQ: Market Quality
LQ: Legal Quality
DQ: Document Quality
RPI: Rensselaer Polytechnic Institute
GE: Generic Electric Co
CT: Computed tomography
2D: Two-dimensional
3D: Three-dimensional
FBP: Filtered backprojection
SPECT: Single photon emission computed tomograph
PET: Positron emission tomography
